# Myocardial Fibrosis and Cardiac Decompensation in Aortic Stenosis

**DOI:** 10.1016/j.jcmg.2016.10.007

**Published:** 2017-11

**Authors:** Calvin W.L. Chin, Russell J. Everett, Jacek Kwiecinski, Alex T. Vesey, Emily Yeung, Gavin Esson, William Jenkins, Maria Koo, Saeed Mirsadraee, Audrey C. White, Alan G. Japp, Sanjay K. Prasad, Scott Semple, David E. Newby, Marc R. Dweck

**Affiliations:** a BHF/Centre for Cardiovascular Science, University of Edinburgh, Edinburgh, United Kingdom; b Department of Cardiovascular Science, National Heart Center, Singapore; c First Department of Cardiology, Poznan University of Medical Sciences, Poznan, Poland; d Royal Brompton Hospital, London, United Kingdom; e Clinical Research Imaging Centre, University of Edinburgh, Edinburgh, United Kingdom

**Keywords:** aortic stenosis, fibrosis, hypertrophy, magnetic resonance imaging, myocardium, T1 mapping, AVR, aortic valve replacement, BNP, brain natriuretic peptide, CMR, cardiac magnetic resonance, cTnI, cardiac troponin I, ECV, extracellular volume, ECG, electrocardiogram, iECV, indexed extracellular volume, IQR, interquartile range, LGE, late gadolinium enhancement, LV, left ventricular, LVH, left ventricular hypertrophy

## Abstract

**Objectives:**

Cardiac magnetic resonance (CMR) was used to investigate the extracellular compartment and myocardial fibrosis in patients with aortic stenosis, as well as their association with other measures of left ventricular decompensation and mortality.

**Background:**

Progressive myocardial fibrosis drives the transition from hypertrophy to heart failure in aortic stenosis. Diffuse fibrosis is associated with extracellular volume expansion that is detectable by T1 mapping, whereas late gadolinium enhancement (LGE) detects replacement fibrosis.

**Methods:**

In a prospective observational cohort study, 203 subjects (166 with aortic stenosis [69 years; 69% male]; 37 healthy volunteers [68 years; 65% male]) underwent comprehensive phenotypic characterization with clinical imaging and biomarker evaluation. On CMR, we quantified the total extracellular volume of the myocardium indexed to body surface area (iECV). The iECV upper limit of normal from the control group (22.5 ml/m^2^) was used to define extracellular compartment expansion. Areas of replacement mid-wall LGE were also identified. All-cause mortality was determined during 2.9 ± 0.8 years of follow up.

**Results:**

iECV demonstrated a good correlation with diffuse histological fibrosis on myocardial biopsies (r = 0.87; p < 0.001; n = 11) and was increased in patients with aortic stenosis (23.6 ± 7.2 ml/m^2^ vs. 16.1 ± 3.2 ml/m^2^ in control subjects; p < 0.001). iECV was used together with LGE to categorize patients with normal myocardium (iECV <22.5 ml/m^2^; 51% of patients), extracellular expansion (iECV ≥22.5 ml/m^2^; 22%), and replacement fibrosis (presence of mid-wall LGE, 27%). There was evidence of increasing hypertrophy, myocardial injury, diastolic dysfunction, and longitudinal systolic dysfunction consistent with progressive left ventricular decompensation (all p < 0.05) across these groups. Moreover, this categorization was of prognostic value with stepwise increases in unadjusted all-cause mortality (8 deaths/1,000 patient-years vs. 36 deaths/1,000 patient-years vs. 71 deaths/1,000 patient-years, respectively; p = 0.009).

**Conclusions:**

CMR detects ventricular decompensation in aortic stenosis through the identification of myocardial extracellular expansion and replacement fibrosis. This holds major promise in tracking myocardial health in valve disease and for optimizing the timing of valve replacement. (The Role of Myocardial Fibrosis in Patients With Aortic Stenosis; NCT01755936)

## Graphical abstract

**Figure:**
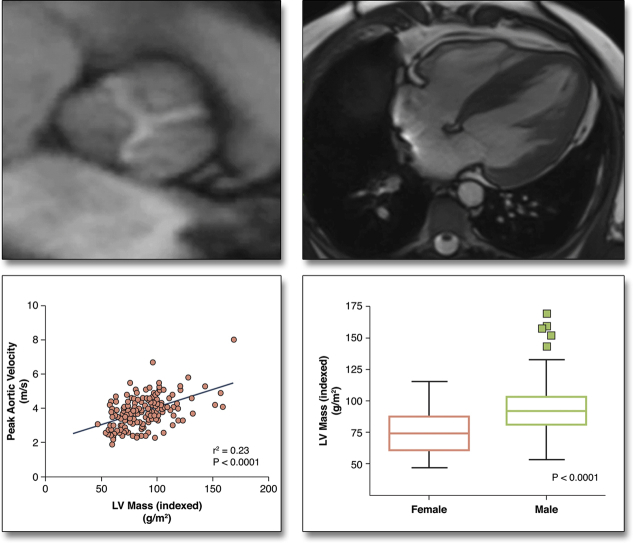

Calcific aortic stenosis is the most common valvular heart condition in the western world and a major public health burden (1). In recent years, the role of left ventricular (LV) remodeling in disease progression, symptom development, and adverse cardiovascular events in aortic stenosis has been increasingly appreciated (2). In the initial phases, the increased afterload imposed by aortic valve narrowing induces adaptive left ventricular hypertrophy (LVH) that acts to maintain wall stress and cardiac output. Ultimately, this process decompensates, and patients transition from hypertrophy to heart failure and the development of symptoms and adverse cardiovascular events 2, 3. This transition often correlates poorly with the severity of aortic valve narrowing and is predominantly driven by myocardial fibrosis and myocyte cell death (4), which is perhaps a consequence of supply–demand mismatch and myocardial ischemia in the hypertrophied myocardium (2). Therefore, there is considerable interest in developing novel biomarkers to detect the early signs of LV decompensation.

Cardiac magnetic resonance imaging (CMR) provides the noninvasive gold standard method for measuring LV wall thickness, mass, volumes, and ejection fraction. Moreover, it is able to detect structural changes in the LV myocardium, including replacement fibrosis with the late gadolinium technique and expansion of the extracellular volume using T1 mapping (5). The latter in part reflects increases in diffuse myocardial fibrosis (a reversible early form of fibrosis) (6) and potential changes in the intravascular compartment. Early studies have suggested that CMR-derived measures of LV mass and replacement myocardial fibrosis are of prognostic significance 7, 8. However, these studies have largely been conducted in small cohorts of patients with end-stage aortic stenosis who were referred to CMR on clinical grounds. Therefore, these findings may have been confounded by referral bias, which limited their applicability and generalizability to the broad population of patients with aortic stenosis. Moreover, comparisons with age- and sex-matched control populations and prognostic T1 mapping studies have been lacking.

We report the largest prospective study to evaluate systematically the usefulness of CMR in patients with aortic stenosis. In particular, we investigated its ability to detect expansion of extracellular volume (ECV) and replacement myocardial fibrosis, and how these are related to other markers of LV decompensation, functional capacity, and clinical outcomes.

## Methods

### Study population

All stable patients with at least mild aortic stenosis (aortic jet velocity ≥2 m/s) who attended the Edinburgh Heart Centre between March 2012 and August 2014 were invited to participate in this prospective observational cohort study. The exclusion criteria were other forms of valvular heart disease (≥ moderate severity), significant co-morbidities with limited life expectancy, contraindications to gadolinium-enhanced CMR, and acquired or inherited nonischemic cardiomyopathies (as assessed by clinical history or ultimately by CMR). In addition, we recruited healthy volunteers from the community with similar demographic characteristics in terms of age and sex, but no history or clinical features consistent with current cardiovascular disease. The study was conducted in accordance with the Declaration of Helsinki and approved by the local research committee. Written informed consent was obtained from all participants.

### Subject characterization

All subjects underwent detailed clinical evaluation including history, physical examination, and electrocardiography. In addition, venous blood samples were obtained for evaluation of biochemistry and cardiac biomarkers of interest.

#### Cardiac biomarkers

Plasma cardiac troponin I (cTnI) concentrations were determined by the ARCHITECT STAT high-sensitivity cTnI assay (Abbot Laboratories, Abbott Park, Illinois) (9). The brain natriuretic peptide (BNP) concentration was determined with Triage BNP assay (Biosite Inc., San Diego, California).

#### 6-min walk test

A 6-min walk test was performed in 156 (94%) patients as an objective measure of functional capacity in our predominantly older adult cohort, many of whom could not perform an exercise tolerance test. Explicit instructions were given to patients asking them to walk as far as possible for 6 min.

#### Echocardiography

Comprehensive transthoracic echocardiography was performed in all patients (iE33, Philips Medical Systems, the Netherlands) by a dedicated research ultrasonographer (A.C.W.) and a cardiologist certified in echocardiography (C.W.L.C.). The severity of aortic stenosis and diastolic function were assessed according to American Society of Echocardiography (ASE) guidelines (Online Appendix).

#### Cardiac magnetic resonance

CMR was performed using a 3-T scanner (MAGNETOM Verio, Siemens AG, Erlangen, Germany). Short-axis cine images were acquired and used to calculate ventricular volumes, mass, and function. Left ventricular hypertrophy (LVH) was defined as LV mass (indexed to body surface area using the Du Bois formula) >95th percentile using age- and sex-specific reference ranges (10). LV longitudinal function was determined by measuring the difference in mitral annular displacement between end-systole and end-diastole (Online Appendix).

Focal replacement fibrosis and ECV expansion were assessed in all patients using late gadolinium enhancement (LGE) and myocardial T1 mapping, respectively. LGE was performed 15 min after administration of 0.1 mmol/kg of gadobutrol (Gadovist, Bayer Pharma AG, Barmen, Germany). The presence of mid-wall myocardial fibrosis was determined qualitatively by 2 independent and experienced operators (M.R.D. and C.W.L.C.), and its distribution was recorded 7, 9.

T1 mapping was performed using the Modified Look-Locker Inversion recovery (11) and a standardized image analysis approach (12). In the short-axis mid-cavity myocardium, 6 standard segments were defined on native T1 maps, and these regions were then copied onto the corresponding 20-min post-contrast maps (OsiriX version 4.1.1, Geneva, Switzerland). Analysis of mid-ventricle segments has been shown to correlate well with analysis of all 17 myocardial segments, is simpler to perform, and avoids partial volume effects in apical segments (12). Segments with mid-wall LGE present were included in this analysis, whereas segments that contained subendocardial, infarct-pattern LGE were excluded. Four commonly used T1 approaches were assessed: native and post-contrast myocardial T1, partition coefficient (lambda), and the ECV fraction. We recently reported the reproducibility of these measures at 3-T (12).

We also investigated a novel marker, the indexed extracellular volume (iECV), which modifies the ECV fraction to act as a measure of the total volume of the extracellular compartment in the left ventricle. It was derived using the formula: ECV fraction × LV end-diastolic myocardial volume normalized to the body surface area.

### Histological validation of myocardial fibrosis

All patients who underwent surgical aortic valve replacement were approached regarding intraoperative myocardial biopsy at the time of surgery. Biopsies were obtained from the basal muscular septum 2 cm below the outflow tract using a Tru-Cut needle (Carefusion, Vernon Hills, Illinois), and then were stained with picrosirius red and analyzed using an automated segmentation tool (Online Appendix). Two blinded and independent observers (A.T.V. and G.E.) analyzed all the specimens, and the interobserver reproducibility was 4.1 ± 2.6%.

### Clinical outcomes

We examined the prognostic value of the different patterns of fibrosis on all-cause mortality as our primary outcome. Patients were followed between March 2012 and September 2015. All deaths were identified through the General Register of Scotland. We also assessed aortic stenosis–related mortality. This was established from the official death certificate and defined as any death in which aortic stenosis was listed as either the primary cause or a contributing factor to that death by the clinical care team.

### Statistical analysis

We assessed the distribution of all continuous variables using the Shapiro-Wilk test and presented them as mean ± SD or median (interquartile range [IQR]). Comparisons were made using the 2-sample *t* test and the Mann-Whitney test where appropriate. We presented all categorical variables as percentages and used the chi-square test for comparison. The relationship between 2 continuous variables was assessed using either Pearson’s r or Spearman’s rho, as appropriate. Potential confounders were adjusted using multivariable linear regression analyses. Time-to-first event survival curves associated with the categories of LV decompensation were estimated using the Kaplan-Meier method and compared with the log-rank test. All statistical analyses were performed using SPSS version 20 (IBM, Armonk, New York) and GraphPad Prism version 6.0 (GraphPad, San Diego, California). A 2-sided p < 0.05 was considered statistically significant.

## Results

### Study population

A total of 203 subjects were recruited: 166 patients with aortic stenosis (peak aortic valve velocity: 3.8 ± 0.90 m/s) and 37 healthy volunteers. These 2 groups were well matched for age, sex, chronic renal impairment, and diabetes. Although a history of hypertension was more common in the aortic stenosis group, blood pressure was well-controlled and similar between the 2 groups at enrollment (Table 1).

**Table 1:** Baseline Characteristics of Patients With Aortic Stenosis and Healthy Volunteers

	Healthy Volunteers (n = 37)	Aortic Stenosis (n = 166)	p Value
Age, yrs	68 (63−74)	69 (63−75)	0.44
Men	24 (65)	115 (69)	0.57
Hypertension	10 (27)	112 (67)	<0.001
Diabetes mellitus	0	25 (15)	—
Coronary artery disease	3 (8)	62 (37)	—
Coronary CTA assessment	13 (35)	21 (13)	—
Invasive coronary angiography	3 (8)	78 (47)	—
Previous PCI	2 (5)	11 (6)	—
Previous CABG	0	8 (5)	—
Atrial fibrillation	0	4 (2)	—
Body mass index, kg/m^2^	27.0 ± 3.6	28.9 ± 4.8	0.02
Body surface area, m^2^	1.86 ± 0.16	1.88 ± 0.19	0.54
NYHA functional class			
I	36 (97)	74 (45)	
II	1 (3)	56 (34)	<0.001
III	—	32 (19)	
IV	—	4 (2)	
6-min walk test, m	430 (400−475)	400 (340−450)	0.001
Systolic blood pressure, mm Hg	148 ± 16	151 ± 21	0.53
Biomarkers			
High sensitivity troponin I concentration, ng/l	3.1 (1.2−7.1)	6.6 (3.8−12.4)	<0.001
Brain natriuretic peptide concentration, pg/ml	9.5 (5.1−20.6)	26.1 (10.7−54.3)	0.001
Echocardiography			
Aortic valve area, cm^2^	2.4 ± 0.6	1.0 ± 0.4	<0.001
Peak aortic jet velocity, m/s	1.4 ± 0.2	3.8 ± 0.9	<0.001
Mean pressure gradient, mm Hg	4.2 ± 1.4	35 ± 19	<0.001
Dimensionless index	0.71 (0.67−0.81)	0.26 (0.22−0.32)	<0.001
Valvulo-arterial impedance, mm Hg/ml/m^2^	4.0 (3.6−4.7)	4.3 (3.6−5.1)	0.38
Mean e', cm/s	7.3 (6.2−8.1)	5.9 (4.9−7.5)	<0.001
Mean E/e' ratio	8.5 (7.0−10.4)	12.6 (10.3−16.9)	<0.001
Mean diastolic dysfunction grade	0.5 ± 0.8	2.0 ± 0.9	<0.001
Cardiac magnetic resonance			
EDVi, ml/m^2^	66 (60−80)	69 (61−78)	0.52
End-systolic volume (indexed), ml/m^2^	23 (19−29)	23 (18−27)	0.40
Stroke volume (indexed), ml/m^2^	44 (40−50)	47 (40−54)	0.16
Systolic ejection fraction, %	65 (62−68)	67 (63−71)	0.02
Longitudinal function, mm	14.8 ± 2.7	12.2 ± 2.9	<0.001
LVMi, g/m^2^	62 (54−71)	88 (73−99)	<0.001
LVMi/EDVi, g/ml	0.92 (0.84−0.99)	1.24 (1.04−1.44)	<0.001
Maximal myocardial wall thickness, mm	7.5 (6.8−8.7)	11.4 (8.8−14.2)	<0.001
Mean myocardial wall thickness, mm	5.6 (5.0−6.3)	7.4 (6.3−9.0)	<0.001
Indexed left atrial volume, ml/m^2^	28 ± 11	36 ± 15	0.01
Mid-wall late gadolinium enhancement	0	44 (27)	—
Native myocardial T1, ms	1,166 ± 27	1,184 ± 42	0.02
20-min post-contrast myocardial T1, ms	645 ± 51	638 ± 46	0.47
Partition coefficient	0.45 ± 0.02	0.46 ± 0.04	0.06
Extracellular volume fraction, %	26.5 ± 1.3	27.7 ± 2.6	0.005
Fibrosis volume, ml	29.9 ± 7.3	44.4 ± 15.1	<0.0001
Indexed extracellular volume, ml/m^2^	16.1 ± 3.2	23.6 ± 7.2	<0.0001

Values are median (interquartile range), n (%), or mean ± SD. Coronary artery disease was defined by previous myocardial infarction, clinical symptoms of angina with documented evidence of myocardial ischemia in the absence of severe aortic stenosis, a >50% luminal stenosis in a major epicardial coronary artery or previous coronary revascularization. All patients with clinical symptoms of angina underwent coronary angiography.

CABG = coronary artery bypass graft; CTA = computed tomography angiography; EDVi = end-diastolic volume (indexed); LVMi = left ventricular mass (index); NYHA = New York Heart Association; PCI = percutaneous coronary intervention.

### Left ventricular hypertrophy

**Figure 1:**
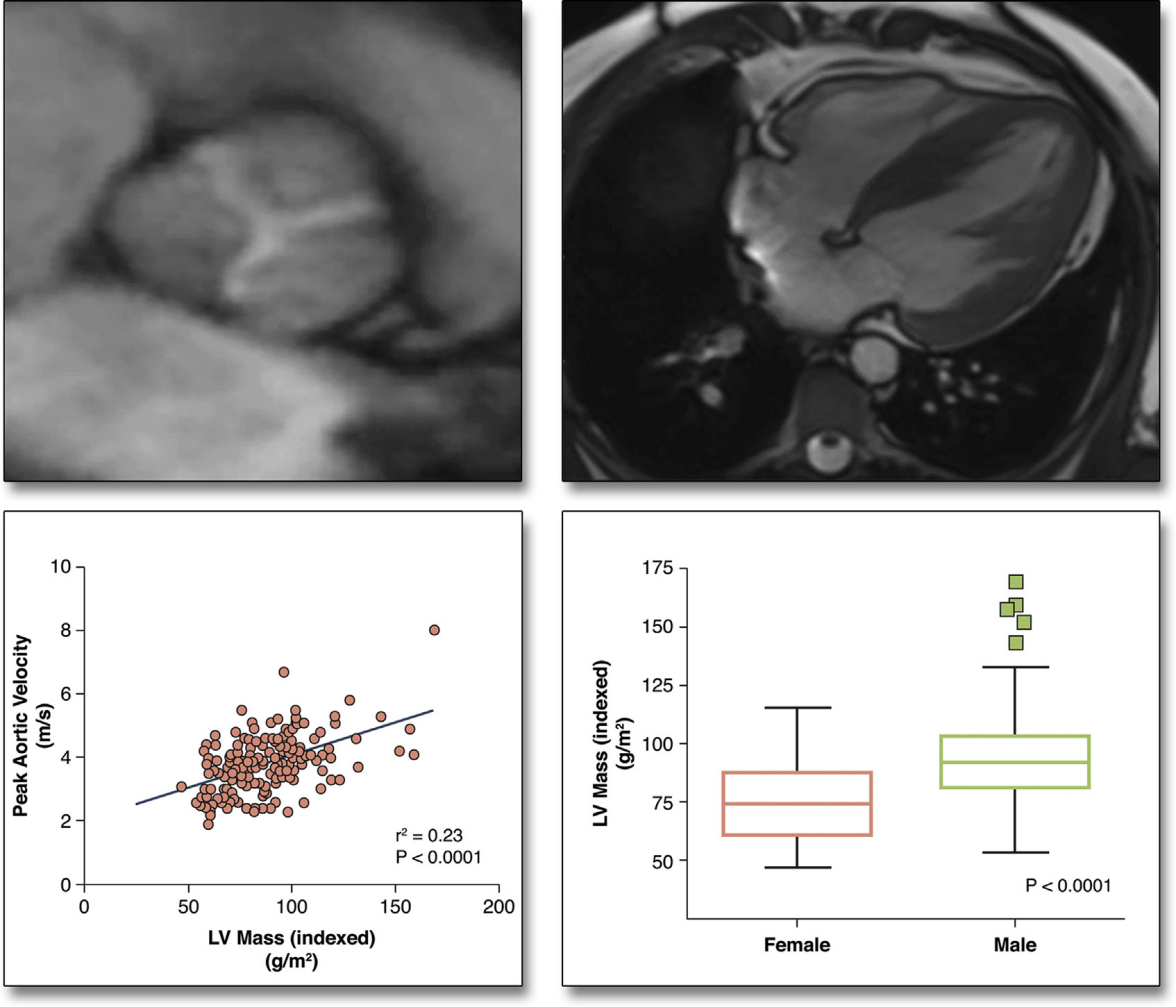
Factors Governing the Magnitude of the Hypertrophic Response in Aortic Stenosis Only a modest correlation between the severity of valve narrowing and the magnitude of the hypertrophic response was observed. The other predictor of left ventricular (LV) mass index on multivariate analysis was sex, with men having more hypertrophy than women.

Although the severity of aortic stenosis correlated positively with LVH (LV mass index: r = 0.48; p < 0.001), it accounted for less than one-quarter (r^2^ = 0.23) of the variance observed (Figure 1). Male sex and aortic stenosis severity were the only independent predictors of LV mass (p < 0.001 for both), independent of systolic blood pressure, age, and coronary artery disease status.

### T1 mapping and extracellular expansion

**Figure 2:**
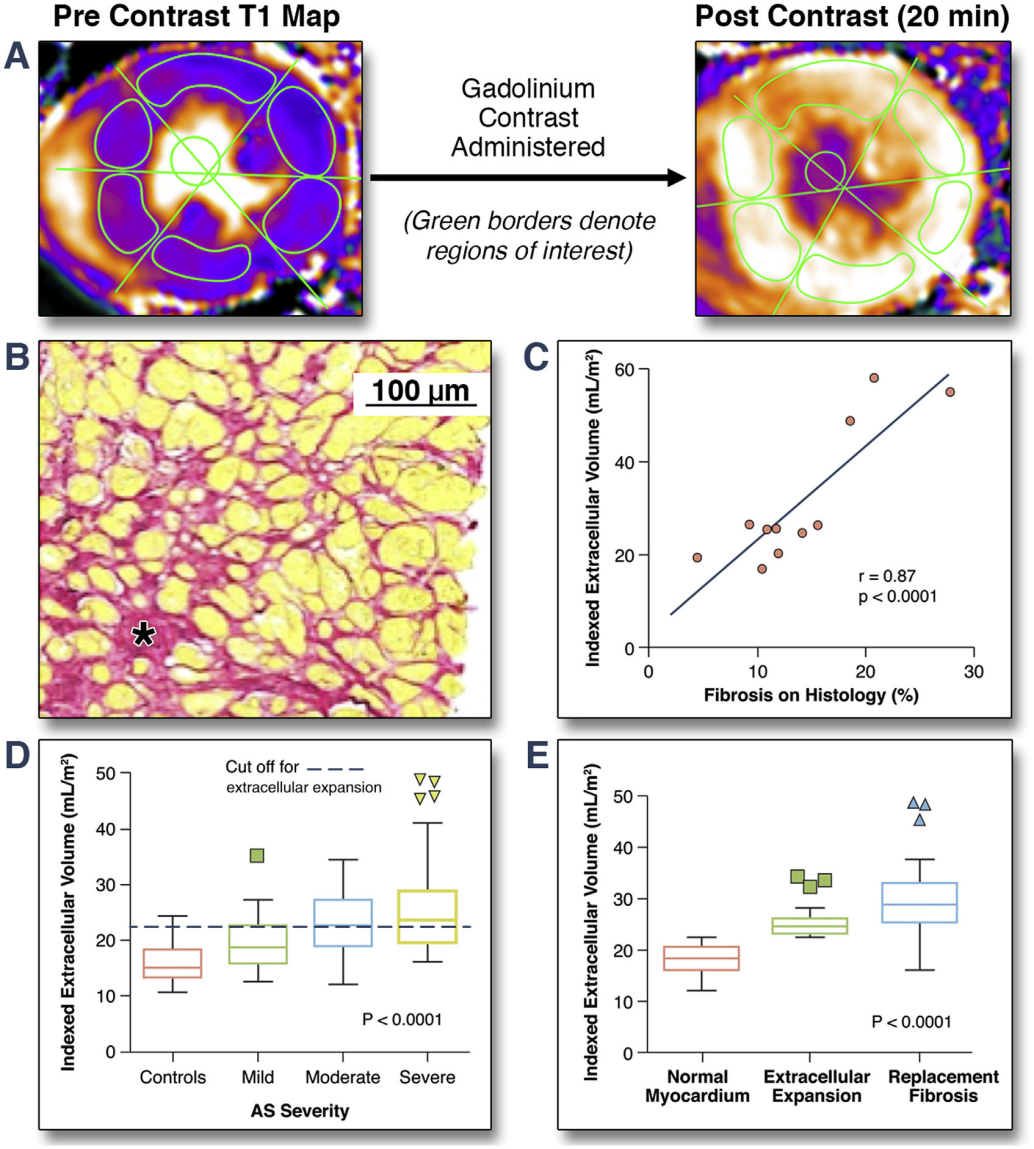
iECV as a Marker of Extracellular Expansion in the Myocardium **(A)** Regions of interest manually drawn onto native and post-contrast T1 maps are used to calculate indexed extracellular volume (iECV). **(B)** Histology from a patient with aortic stenosis (AS) with areas of diffuse fibrosis stained with picrosirus red. **(C)** Excellent correlation between iECV and diffuse myocardial fibrosis on histology. **(D)** iECV provided good discrimination between disease states. **(E)** iECV values were higher in patients with replacement fibrosis than patients with normal myocardium or extracellular expansion.

Myocardial biopsies were obtained in 11 of 37 patients who underwent surgical aortic valve replacement. Strong correlations were observed between the amount of myocardial fibrosis on histology and T1 mapping parameters (native T1: r = 0.76; p = 0.007; lambda: r = 0.82; p = 0.002; ECV fraction: r = 0.70; p = 0.016; and iECV: r = 0.87; p < 0.001) (Figure 2), with the exception of post-contrast myocardial T1 (r = 0.01; p = 0.98). Indexed LV mass also correlated well with histological fibrosis (r = 0.83; p < 0.001).

Compared with the healthy volunteers, patients with aortic stenosis had increased diffuse myocardial fibrosis, with iECV providing the best discrimination between cases and control subjects (23.6 ± 7.2 ml/m^2^ vs. 16.1 ± 3.2 ml/m^2^; p < 0.0001) (Table 1 and Figure 2). Moreover, of all the T1 measures, only the iECV demonstrated a progressive increase across patients with mild, moderate, and severe aortic stenosis (19.6 ± 4.6 ml/m^2^ vs. 22.9 ± 5.4 ml/m^2^ vs. 25.5 ± 8.1 ml/m^2^, respectively; p < 0.001) (Table 2). Notably, the ECV fraction did not vary with aortic stenosis severity (as measured by peak aortic valve velocity; p = 0.30 (Online Table 1) and showed a high degree of overlap between cases and control subjects (26.5 ± 1.4% vs. 27.7 ± 2.6%; p = 0.007).

**Table 2:** Cardiovascular Magnetic Resonance Measures of Myocardial Fibrosis and Functional Status by Severity of Aortic Stenosis

	Mild (n = 34)	Moderate (n = 45)	Severe (n = 87)	p Value
Age, yrs	67 (56−75)	72 (66−77)	71 (65−76)	0.048
Men	20 (59)	32 (71)	63 (72)	0.33
EDVi, ml/m^2^	69 (60−77)	68 (64−81)	69 (61−79)	0.72
End-systolic volume (indexed), ml/m^2^	24 (19−26)	23 (20−27)	22 (17−27)	0.87
Stroke volume (indexed), ml/m^2^	47 (39−52)	47 (42−56)	47 (40−54)	0.56
Systolic ejection fraction, %	67 (63−69)	66 (63−70)	67 (63−72)	0.65
Longitudinal function, mm	13.6 ± 2.4	13.2 ± 2.9	11.2 ± 2.8	<0.001
LVMi, g/m^2^	71 (61−86)	87 (74−98)	93 (80−104)	<0.001
LVMi/EDVi, g/ml	1.08 ± 0.20	1.21 ± 0.23	1.36 ± 0.28	<0.001
Maximal myocardial wall thickness, mm	8.2 ± 2.1	11.1 ± 3.3	13.4 ± 3.4	<0.001
Mean myocardial wall thickness, mm	5.9 ± 1.1	7.3 ± 1.6	8.7 ± 1.9	<0.001
Patients with LVH	6 (17)	24 (53)	59 (68)	<0.001
Native myocardial T1, ms	1,170 ± 30	1,180 ± 37	1,192 ± 46	0.02
20-min post-contrast myocardial T1, ms	637 ± 45	643 ± 48	636 ± 45	0.73
Partition coefficient	0.466 ± 0.03	0.466 ± 0.04	0.466 ± 0.05	0.07
ECV fraction, %	27.8 ± 2.5	27.5 ± 2.0	27.8 ± 3.0	0.79
iECV, ml/m^2^	19.6 ± 4.6	22.9 ± 5.4	25.5 ± 8.1	<0.001
Extracellular expansion (iECV ≥22.5 ml/m^2^)	9 (26)	23 (51)	47 (54)	0.021
Mid-wall late gadolinium enhancement	2 (5.9)	14 (31)	28 (32)	0.008
Diastolic function (E/e')	11.1 (8.0−14.2)	12.2 (10.1−16.4)	13.5 (11.4−18.6)	0.009
Natural log (hs troponin I)	1.25 (0.72−1.55)	1.76 (1.33−2.34)	2.16 (1.59−2.81)	<0.0001
6-min walk test, m	420 (363−448)	400 (340−450)	390 (320−440)	0.05

Values are median (interquartile range), n (%), or mean ± SD.

ECV = extracellular volume; LVH = left ventricular hypertrophy; other abbreviations as in Tables 1 and 2.

We explored iECV in greater detail, dividing our entire patient cohort into tertiles of iECV (Table 3). Using this approach, a steady increase across the tertiles was observed for each of the following markers of disease severity and LV decompensation: indexed LV mass, peak aortic valve velocity, plasma high-sensitivity cTnI concentrations, serum BNP concentrations, diastolic dysfunction, longitudinal systolic dysfunction, and the proportion of patients with mid-wall fibrosis (p < 0.05 for all). Similar results were obtained using tertiles of the ECV fraction (Online Table 1), but by comparison, tertiles of LV mass index were less discriminatory, with no differences in diastolic function nor in serum BNP concentrations across these groups (both p > 0.05) (Online Table 2).

**Table 3:** Progressive Increase in Markers of LV Hypertrophy and Decompensation With Increasing iECV Stratified Into Tertiles

	Tertile 1 (n = 54)	Tertile 2 (n = 54)	Tertile 3 (n = 53)	p Value
Age, yrs	70 (63−75)	70 (65−70)	72 (64−78)	0.30
Men	27 (50)	42 (78)	43 (81)	0.0006
Echocardiography				
Peak aortic jet velocity, m/s	3.45 ± 0.78	3.77 ± 0.81	4.25 ± 0.96	<0.0001
Aortic valve area, cm^2^	1.01 ± 0.37	0.95 ± 0.36	0.90 ± 0.35	0.32
Mean AV pressure gradient, mm Hg	27.6 ±12.7	32.7 ± 15.0	42.6 ± 23.7	<0.0001
Mild aortic stenosis	19	12	3	
Moderate aortic stenosis	13	17	15	
Severe aortic stenosis	22	25	35	
Valvulo-arterial impedance, mm Hg/ml/m^2^	4.4 ± 1.1	4.0 ± 1.0	3.8 ± 1.0	0.019
Mean e', cm/s	6.9 ± 2.0	6.4 ± 1.7	5.4 ± 1.8	<0.0001
Mean E/e' ratio	11.6 (9.8−14.4)	12.4 (9.3−16.5)	14.3 (11.9−19.2)	0.02
Mean diastolic dysfunction grade	1.5 ± 1.0	2.0 ± 0.8	2.5 ± 0.7	<0.0001
CMR				
LVMi, g/m^2^	68 ± 9	88 ± 9	110 ± 19	<0.0001
Ejection fraction, %	68 (63−71)	67 (64−73)	66 (61−71)	0.44
Longitudinal function, mm	13.0 ± 2.7	12.8 ± 2.7	11.0 ± 3.0	0.0004
Mid-wall fibrosis	2 (4)	6 (11)	36 (68)	0.0001
Indexed left atrial volume, ml/m^2^	29 ± 13	36 ± 14	38 ± 13	0.004
Biomarkers				
Natural log (hs troponin I)	1.3 (0.8−1.6)	2.1 (1.5–2.4)	2.5 (1.9−3.3)	<0.0001
Natural log (BNP)	2.8 (1.9−3.5)	3.1 (2.4−3.9)	4.0 (2.8−4.7)	<0.0001
Functional status				
6-min walk test, m	410 (345−445)	410 (358−453)	385 (295−443)	0.09
NYHA functional class, %				
I	27 (50)	23 (43)	24 (45)	
II	20 (37)	19 (35)	15 (28)	
III	6 (11)	12 (22)	11 (21)	
IV	1 (2)	0 (0)	3 (6)	
Outcomes				
All-cause mortality	2	2	10	—
Mortality rate (per 1,000 patient-years)	12	12	72	0.005
Aortic stenosis-related mortality	0	2	8	—

Values are median (interquartile range), n (%), or mean ± SD. Five patients had insufficient data to calculate the indexed extracellular volume.

AV = aortic valve; BNP = brain natriuretic peptide; CMR = cardiac magnetic resonance; hs = high-sensitivity; other abbreviations as in Table 1.

### Replacement myocardial fibrosis

Replacement mid-wall fibrosis, as assessed by LGE, was present in 44 (27%) patients with aortic stenosis but in none of the healthy volunteers. We examined the association between mid-wall myocardial fibrosis and the severity of aortic stenosis (Table 2). Although patients with mid-wall fibrosis had more severe aortic stenosis compared with those without (peak aortic valve velocity: 4.1 m/s; IQR: 3.7 to 4.6 m/s vs. 3.8 m/s; IQR: 3.4 to 4.6 m/s, respectively; p = 0.001), this difference was small and unlikely to be of any clinical significance. In contrast, patients with mid-wall fibrosis demonstrated a marked 30% increase in LV mass indicative of an advanced hypertrophic response (LV mass index 107 ± 24 g/m^2^ vs*.* 82 ± 16 g/m^2^, respectively; p < 0.001). LV mass index was independently associated with mid-wall myocardial fibrosis in those with hypertrophy (odds ratio: 1.09, 95% confidence interval: 1.04 to 1.14; p < 0.001) after adjusting for aortic stenosis severity, age, sex, and systolic blood pressure.

### Relationship between myocardial ECV and replacement fibrosis

It has been suggested that replacement fibrosis represents the irreversible final stage of diffuse interstitial fibrosis and extracellular expansion. Consistent with this hypothesis, patients with replacement mid-wall fibrosis had evidence of increased ECV on T1 mapping compared with patients without (iECV: 32.0 ml/m^2^; IQR: 29.1 to 34.9 ml/m^2^ vs. 21.5 ml/m^2^; IQR: 20.6 to 22.4 ml/m^2^; p < 0.0001; ECV fraction: 29.1 ± 2.4% vs. 26.9 ± 2.1%; p < 0.001). The iECV was independently associated with mid-wall fibrosis after adjusting for age, sex, severity of aortic stenosis, and even LV mass (odds ratio: 1.22; 95% confidence interval: 1.11 to 1.35; p < 0.001). Similar associations were observed using the ECV fraction.

### Categorization of LV decompensation

**Figure 3:**
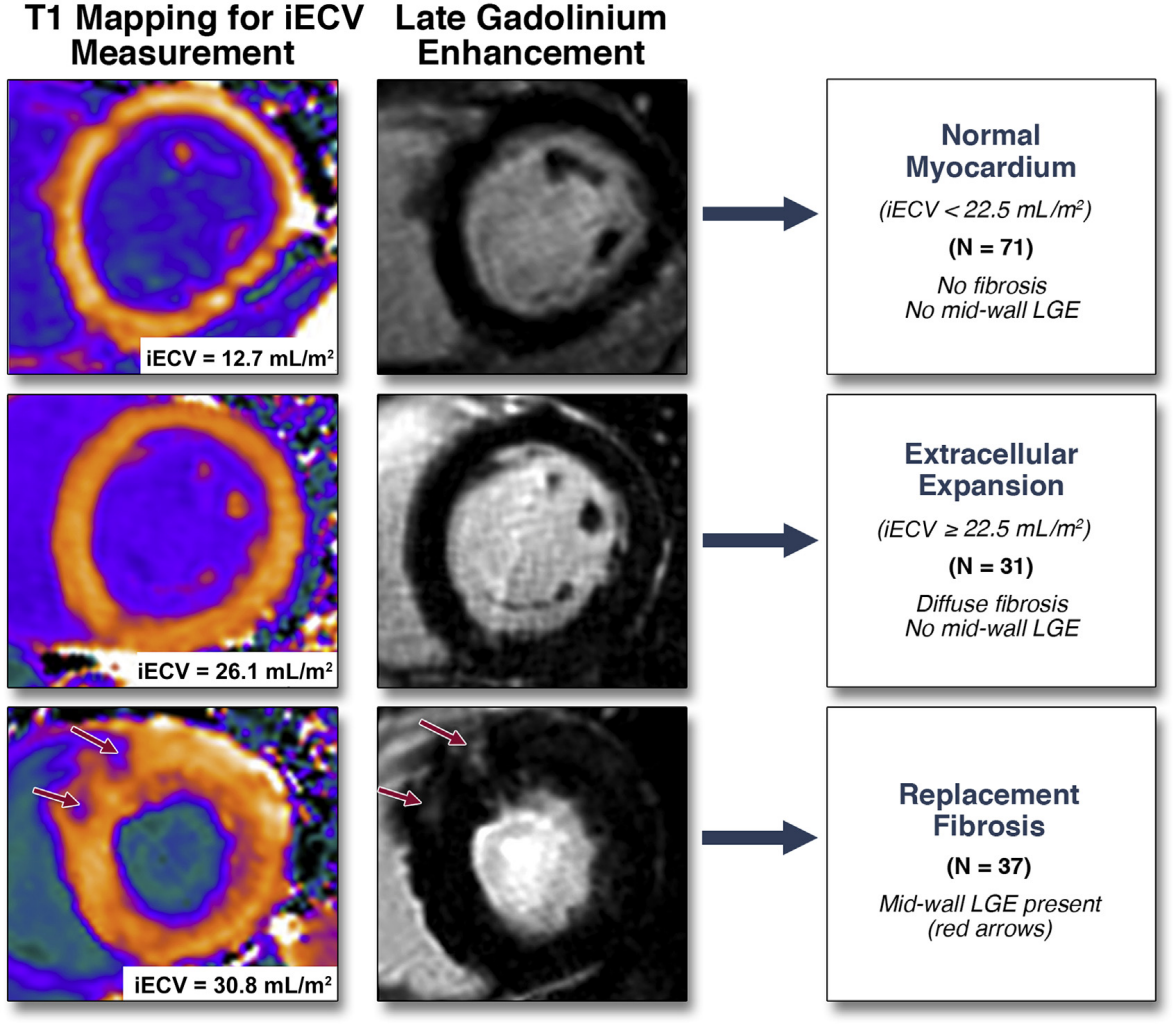
CMR Categorization of Myocardial Fibrosis in Aortic Stenosis Patients with aortic stenosis were categorized into 3 groups based upon cardiac magnetic resonance (CMR) assessments of fibrosis. iECV = indexed extracellular volume; LGE = late gadolinium enhancement.

We proceeded to categorize patients into 3 groups according to our CMR measures of myocardial fibrosis: normal myocardium, extracellular expansion, and replacement mid-wall fibrosis (Figure 3). The upper limit of normal for iECV in the healthy volunteers (defined by 2 SDs above the mean, 22.5 ml/m^2^) was used to define expansion of the extracellular myocardium. Values below this threshold defined normal myocardium. This categorization was then validated in the 11 patients who underwent myocardial biopsy, with the percentage fibrosis on histology increasing progressively across the 3 groups (normal myocardium: 8.9 ± 4.0% vs. extracellular expansion: 12.4 ± 2.5% vs. replacement fibrosis: 22.4 ± 4.9%; p < 0.004) (Table 4).

**Table 4:** Characteristics of Patients Stratified According to iECV Thresholds and Presence of Mid-Wall Late Gadolinium Enhancement

	Normal Myocardium (n = 71)	Extracellular Expansion (n = 31)	Replacement Fibrosis (n = 37)	p Value
Age, yrs	70 (63−75)	70 (63−75)	71 (65−78)	0.59
Sex (male = 1)	0.56 (0.44−0.68)	0.81 (0.67−0.96)	0.76 (0.62−0.90)	0.023
Hypertension	48 (68)	20 (65)	22 (59)	0.70
Diabetes	8 (11)	7 (23)	2 (5)	0.09
Body mass index, kg/m^2^	28.2 ± 4.5	28.9 ± 4.6	29.3 ± 4.3	0.44
Echocardiography				
Peak aortic jet velocity, m/s	3.53 ± 0.82	3.79 ± 1.0	4.23 ± 0.92	<0.001
Aortic valve area, cm^2^	0.98 (0.73−1.18)	0.88 (1.2−0.7)	0.83 (0.73−0.91)	0.049
Mean AV pressure gradient, mm Hg	29.1 ± 14.2	34.8 ± 21.1	41.1 ± 23.5	0.007
Mild AS	24	7	2	
Moderate AS	18	10	11	
Severe AS	29	14	24	
Valvulo-arterial impedance, mm Hg/ml/m^2^	4.1 ± 1.0	3.9 ± 0.9	4.0 ± 1.0	0.46
Mean e', cm/s	6.7 ± 2.0	6.5 ± 1.8	5.2 ± 1.4	0.0004
Mean E/e' ratio	13.1 ± 7.7	13.2 ± 4.8	16.5 ± 6.5	0.04
Mean diastolic dysfunction grade	1.5 ± 0.9	2.0 ± 0.9	2.7 ± 0.5	<0.0001
CMR				
LVMi, g/m^2^	73 ± 11	96 ± 11	107 ± 25	<0.0001
Relative wall thickness	0.60 ± 0.12	0.61 ± 0.09	0.67 ± 0.11	0.018
ECVi, ml/m^2^	18.3 ± 2.5	25.4 ± 3.1	30.4 ± 8.2	<0.0001
Ejection fraction, %	68 (63−71)	66 (64−71)	67 (64−72)	0.94
Longitudinal systolic function, mm	13.2 ± 2.6	12.5 ± 2.4	11.2 ± 3.1	0.002
Indexed left atrial volume, ml/m^2^	31 ± 13	37 ± 11	38 ± 15	0.027
Biomarkers				
Natural log (hs troponin I)	1.43 ± 0.96	2.02 ± 0.93	2.60 ± 0.90	<0.0001
Natural log (BNP)	2.95 ± 1.00	3.06 ± 0.96	3.41 ± 1.10	0.12
Functional status				
6-min walk test, m	406 ± 74	385±95	359 ± 138	0.08
NYHA functional class				
I	33 (46)	18 (58)	17 (46)	
II	27 (38)	6 (19)	12 (32)	
III	11 (15)	7 (23)	5 (14)	
IV	0 (0)	0 (0)	3 (8)	
Outcomes				
All-cause mortality	2	4	8	—
All-cause mortality rate (per 1,000 patient-yrs)	8	36	71	0.009
AS-related mortality, n	0	4	6	—
AS-related mortality rate (per 1,000 patient-yrs)	0	36	52	0.0045

Patients Undergoing Myocardial Biopsy	Normal Myocardium on CMR (n = 3)	Extracellular Expansion on CMR (n = 5)	Replacement Fibrosis on CMR (n = 3)	p Value
Histological fibrosis, %	8.9 ± 4.0	12.4 ± 2.5	22.4 ± 4.9	0.004
Aortic valve area, cm^2^	0.57 ± 0.10	0.94 ± 0.29	0.81 ± 0.44	0.29
Peak aortic jet velocity, m/s	4.6 (4.4−5.1)	4.5 (4.0−5.9)	4.9 (4.1−8.0)	0.63
LVMi, g/m^2^	76 ± 15	98 ± 4	162 ± 6	<0.0001
Native myocardial T1, ms	1,189 ± 23	1,183 ± 16	1,277 ± 15	0.0002
Post-contrast myocardial T1, ms	676 ± 45	615 ± 24	672 ± 84	0.22
Partition coefficient	0.43 ± 0.05	0.47 ± 0.02	0.55 ± 0.02	0.008
ECV fraction, %	25.3 ± 3.1	27.3 ± 1.4	32.2 ± 1.9	0.019
iECV, ml/m^2^	18.8 ± 1.9	25.6 ± 0.7	49.6 ± 4.8	<0.0001

Values are median (interquartile range), n (%), or mean ± SD.

Abbreviations as in Tables 1, 2, and 3.

**Figure 4:**
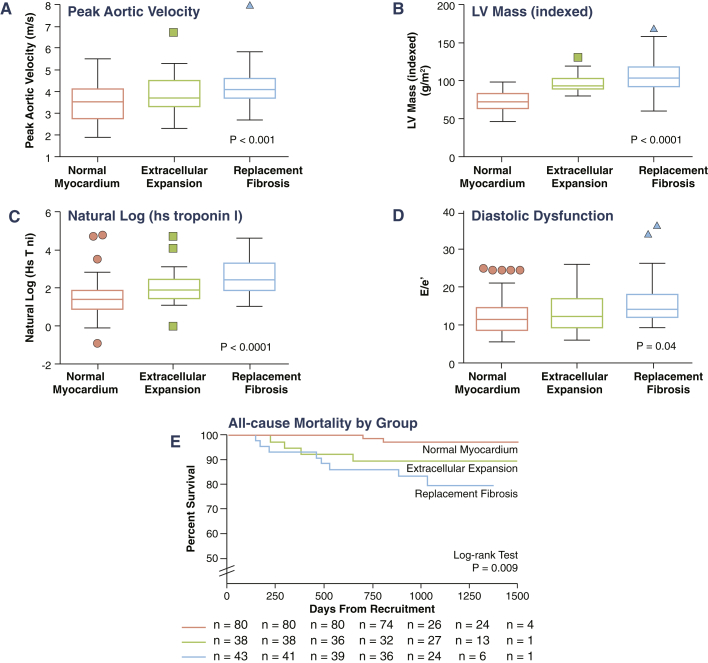
Progressive LV Decompensation on Moving From Normal Myocardium to Extracellular Expansion to Replacement Fibrosis On moving from normal myocardium to extracellular expansion and then replacement fibrosis, there was a stepwise increase in the following measures: **(A)** the severity of valve narrowing; **(B)** the degree of hypertrophy; **(C)** myocardial injury; **(D)** left ventricular (LV) performance; and **(E)** all-cause-mortality. hsTni = high-sensitivity troponin I concentration.

In the larger imaging cohort of patients with aortic stenosis (after exclusion of patients with an infarct pattern of LGE, n = 22, or incomplete T1 mapping data, n = 5), 71 patients had normal myocardium (iECV <22.5 ml/m^2^). These patients had largely mild-to-moderate aortic stenosis, a mild hypertrophic response, minimal cardiac injury, and good LV performance (Table 4, Figures 3 and 4). Thirty-one patients had extracellular expansion (iECV ≥22.5 ml/m^2^), with values for aortic stenosis severity, LV mass, myocardial injury, diastolic function, and longitudinal systolic function that were intermediate between patients with normal myocardium and replacement fibrosis. Finally, 37 patients had evidence of replacement myocardial fibrosis on LGE. These patients were confirmed as having the most severe aortic stenosis, LVH, myocardial injury, and impairment in LV performance (Table 4). Compared with patients with extracellular expansion, they had even higher iECV values (30.4 ± 8.2 ml/m^2^ vs. 25.4 ± 3.1 ml/m^2^; p < 0.0001) (Figure 2), whereas compared with patients with normal myocardium, they had increased serum BNP concentrations (16.7 pg/ml; IQR: 6.1 to 36.0 pg/ml vs. 34.4 pg/ml; IQR: 10.5 to 76.2 pg/ml, respectively, p = 0.026) and impaired functional capacity (6-min walk test: 405 ± 74 m vs. 359 ± 138 m, respectively; p = 0.03). Both mid-wall fibrosis and the ECV fraction were predictors of functional capacity independent of age, sex, LV mass, and peak velocity (Table 5). These findings were unchanged when patients with mild aortic stenosis were excluded from the analysis (Online Table 3).

**Table 5:** Univariable and Multivariable Linear Regression Analysis to Examine the Association of Fibrosis Assessments With Functional Status

	Univariable Analysis		Multivariable Analysis			
	Relative Change in 6-Min Walk (95% CI)	p Value	Model 1 (ECV Fraction)		Model 2 (Mid-Wall Fibrosis)	
			Relative Change in 6-Min Walk (95% CI)	p Value	Relative Change in 6-Min Walk (95% CI)	p Value
Age ≥70 yrs	−50.3 (−83.0 to −17.6)	0.003	−41.4 (−74.5 to −8.36)	0.01	−50.3 (−83.0 to −17.7)	0.003
Men	−0.81 (−37.7 to 36.1)	0.97	−19.9 (−61.3 to 21.6)	0.35	−8.88 (−48.5 to 30.7)	0.66
Peak aortic jet velocity, m/s	−12.25 (−30.6 to 6.14)	0.19	−14.9 (−35.4 to 5.73)	0.16	−11.9 (−32.4 to 8.64)	0.26
LVMi, g/m^2^	−0.22 (−1.00 to 0.56)	0.57	0.62 (−0.44 to 1.68)	0.25	0.45 (−0.62 to 1.52)	0.41
ECV fraction, %	−9.09 (−15.4 to −2.81)	0.005	−9.77 (−17.0 to −2.58)	0.01	—	—
Presence of mid−wall fibrosis	−40.9 (−78.5 to −3.24)	0.03	—	—	−45.6 (−89.1 to −2.11)	0.04

CI = confidence interval; other abbreviations as in Tables 2 and 3.

### Clinical outcomes

Participants were followed up for an average of 2.9 ± 0.8 years during which a total of 14 patients with aortic stenosis died: 2 with normal myocardium, 4 with extracellular expansion and 8 with replacement fibrosis. Unadjusted all-cause mortality rates rose progressively across the groups (8 deaths/1,000 patient-years vs. 36 deaths/1,000 patient-years vs. 71 deaths/1,000 patient-years; log-rank test: p = 0.009 (Table 4, Figure 4). AS-related mortality also increased in a stepwise fashion (0 deaths/1,000 patient-years vs. 36 deaths/1,000 patient-years vs. 52 deaths/1,000 patient-years; p = 0.0045) with no AS-related deaths in the normal myocardium group. Tertiles of ECV fraction (p = 0.0006) (Online Table 1) and iECV (p = 0.005) (Table 3) also displayed prognostic ability in this unadjusted analysis but no difference in mortality was observed across tertiles of the indexed LV mass (p = 0.23) (Online Table 2).

## Discussion

This is the largest prospective CMR study to systematically evaluate both extracellular expansion and replacement fibrosis in the myocardium of patients with aortic stenosis and healthy control subjects. Both measures are increased in aortic stenosis, but are only weakly associated with the severity of valve narrowing. In contrast, they demonstrate a close association with the magnitude of the hypertrophic response, the presence of LV dysfunction, the functional capacity of the patient, and, ultimately, clinical outcome. We believe these findings demonstrate that the structural changes in the LV myocardium are as important a consideration as the severity of the valvular disease itself. Based on these results, we propose that patients with aortic stenosis be categorized into 3 groups—those with normal myocardium, extracellular expansion, and replacement myocardial fibrosis. We believe this classification has major potential in the early detection of subclinical ventricular decompensation in aortic stenosis and ultimately may be able to guide decisions regarding the timing of aortic valve replacement.

Our data demonstrated an association between the severity of valve narrowing and the degree of hypertrophy in aortic stenosis. However, this only explained approximately one-quarter of the observed variance in LV mass, confirming that the hypertrophic response in aortic stenosis cannot be accurately predicted from the degree of valve narrowing alone and should be assessed independently.

T1 mapping techniques (ECV fraction and iECV) can provide an assessment of myocardial ECV expansion. Potentially, this can reflect increased myocardial fibrosis, myocardial infiltration, or expansion in the intravascular compartment (13). In aortic stenosis, myocardial fibrosis has been established pathologically as a key process that drives the transition from hypertrophy to heart failure (4). Moreover, we and others have observed a close correlation between these parameters and histological assessments of myocardial fibrosis 5, 14, 15, 16. However, there is some debate as to whether T1 mapping can provide direct assessment of the myocardium because of recent evidence that indicated that increased intravascular volume may also influence native T1 values 17, 18. Pressure overload conditions such as aortic stenosis are associated with reduced capillary density (19) and myocardial ischemia (20). Mahmod et al. (17) recently suggested that this ischemia might result in coronary vasodilatation and increased intravascular volume potentially contributing to increased native T1. Although confirmation of this interesting hypothesis is required, it may be that T1 values are also related to myocardial ischemia that is believed to trigger fibrosis and the transition from hypertrophy to heart failure. Regardless, T1 mapping remains at the very least a useful surrogate of myocardial fibrosis and LV decompensation in aortic stenosis.

Controversy remains as to the optimal T1 image analysis strategy 12, 13, 21. Consistent with previous research (12), both native T1 and the ECV fraction demonstrated major overlap with values in control groups and little difference among patients with mild, moderate, and severe aortic stenosis (Table 2). We sought to tackle this issue by developing a novel parameter, the iECV, which provides an assessment of the total ECV in the myocardium. This effectively combines the prognostic information provided by the ECV fraction with the improved discrimination between groups associated with indexed LV mass into a single measure (Table 3, Online Tables 1 and 2).

iECV demonstrated good correlation with histological assessment of fibrosis burden. Moreover, there was a clear stepwise increase across tertiles of iECV in each of the different clinical and imaging measures of LV decompensation, as well as clinical outcomes, supporting iECV as a marker of decompensation. Finally, iECV provided the best discrimination among disease states, being the only T1 measure to differentiate among patients with mild, moderate, and severe aortic stenosis. In combination, iECV would therefore appear to provide the most useful marker of LV decompensation in aortic stenosis with advantages compared with both the ECV fraction and LV mass in isolation.

How do extracellular expansion and diffuse fibrosis relate to the development of replacement fibrosis as detected using mid-wall LGE? In agreement with previous studies 7, 8, regions of mid-wall LGE were observed in 27% of our patients, with approximately two-thirds with severe aortic stenosis and one-third with moderate aortic stenosis. Importantly, patients with mid-wall replacement fibrosis also had marked increases in iECV as a surrogate for diffuse fibrosis in their remote myocardium. Indeed, iECV was an independent predictor of the presence of mid-wall LGE. This was confirmed by our histological data and suggests that replacement fibrosis does not occur until the end stages of myocardial matrix remodeling and is preceded by an intermediate stage of extracellular expansion reflecting increasing diffuse fibrosis. Longitudinal studies using serial CMR imaging are required to confirm this hypothesis.

Using our CMR assessments, we categorized our patients into 3 stages of LV decompensation. We used iECV to differentiate patients with normal myocardium from those with extracellular expansion and then LGE to define replacement fibrosis (Figure 3). Across these groups, patients had advancing LVH, histological fibrosis, myocyte cell injury, diastolic dysfunction, and longitudinal systolic dysfunction, which suggested progressive, subclinical LV decompensation. Most importantly, there was a steady decline in prognosis, with unadjusted all-cause mortality rates quadrupling from the normal myocardium groups to the extracellular expansion groups and more than doubling again in those with replacement fibrosis. Moreover, these groups also predicted aortic stenosis−related deaths on unadjusted analysis, with no aortic stenosis−related deaths occurring in the normal myocardium group. More simple categorization using LV mass was less discriminatory and not of prognostic value (Online Table 2).

Our categorization holds promise as a means of monitoring the development of LV decompensation and helping to optimize the timing of aortic valve replacement. Currently, the development of symptoms guides the need for surgery. However, symptoms are frequently difficult to assess in older adult patients with multiple co-morbidities. Objective imaging assessments that monitor the changes in myocardial structure that are themselves responsible for progressive LV decompensation are therefore potentially attractive 2, 3. This is the first study to describe iECV in aortic stenosis, so that confirmation of our findings in larger studies with longer follow-up is required. However, we presented the fourth separate cohort to demonstrate the adverse prognosis associated with mid-wall LGE in aortic stenosis 7, 8, 22 and demonstrated its association with patient functional capacity, LV performance, and multiple other parameters of LV decompensation. These data have now led to the EVOLVED (Early Valve Replacement guided by Biomarkers of Left Ventricular Decompensation in Asymptomatic Patients with Advanced Aortic Stenosis) study. This multicenter, randomized controlled trial will begin enrollment next year and assess whether early valve intervention in patients with asymptomatic severe aortic stenosis and mid-wall fibrosis on CMR improves clinical outcomes compared with standard care.

### Study limitations

There were insufficient deaths to perform multivariate analysis. Studies with longer follow-up are required to confirm whether iECV is of independent prognostic value and to assess the contribution of the intravascular volume to T1 mapping values. Finally, although similar to previous studies 14, 15, 16, the number of patients who agreed to intraoperative myocardial biopsy was modest, which perhaps reflected the invasive nature of this assessment.

## Conclusions

CMR can detect progressive fibrosis in aortic stenosis and can be used to categorize patients with normal myocardium, extracellular expansion, or replacement fibrosis. Across these groups, there was a stepwise increase in myocardial injury, fibrosis, LV dysfunction, and unadjusted mortality that was consistent with progressive ventricular decompensation. This categorization may be able to track the transition of hypertrophy to heart failure in patients with aortic stenosis.

Perspectives **COMPETENCY IN MEDICAL KNOWLEDGE:** Decompensation of the hypertrophic response in aortic stenosis is driven by progressive myocardial fibrosis and the associated expansion in the ECV. Changes in the LV myocardium can be tracked using CMR, which can be used to categorize patients into 3 groups: normal myocardium, extracellular expansion, and replacement fibrosis. There is evidence of increasing myocyte injury, left ventricular dysfunction, functional impairment, and all-cause mortality across these groups.

**TRANSLATIONAL OUTLOOK:** This categorization holds promise in tracking the transition from hypertrophy to heart failure in aortic stenosis and in identifying the optimal timing of aortic valve replacement. Prospective randomized controlled studies are required to investigate this further.
