# Supplementary material for: Myocardial Fibrosis and Cardiac Decompensation in Aortic Stenosis
Source: JACC Cardiovasc Imaging. 2017 Nov;10(11):1320–33. doi: 10.1016/j.jcmg.2016.10.007 (PMC5683736; doi:10.1016/j.jcmg.2016.10.007)
Supplement: Online Appendix and Online Tables 1–3 [file mmc1.docx]

**SUPPLEMENTARY APPENDIX**

**METHODS**

*Echocardiography*

Comprehensive assessment of aortic stenosis severity was performed. The left ventricular outflow tract diameter was measured in the parasternal long-axis view, at the insertion of the aortic cusps from the inner edge of the septal endocardium to the inner edge of the anterior mitral leaflet in mid-systole. Left ventricular outflow tract velocity-time integral was measured in the apical 5-chamber view using pulsed-wave Doppler just proximal to the aortic valve, with care taken to obtain a laminar spectral tracing. The peak aortic jet velocity and mean transvalvular gradient were derived from the aortic valve velocity-time integral, using continuous-wave Doppler. The highest aortic jet velocity and mean transvalvular gradient were determined in multiple acoustic windows using both standard S51 and D2cwc probes (Philips Medical Systems, Best, the Netherlands), and corroborated between the 2 operators. The mean of 3 readings (5 if the patient had atrial fibrillation) was recorded. Aortic valve area was calculated using the continuity equation.

Diastolic function was assessed using multiple parameters: E/A ratio, mean e’, E/e’, LA volume (measured using CMR) and diastolic dysfunction grade. Trans-mitral early (E) and late diastolic velocities, as well as, deceleration time of early filling velocity were measured at the tips of the mitral valve leaflets using pulsed-wave Doppler. The mean early diastolic velocities of the medial and lateral mitral annulus (e’) were measured using pulsed-wave tissue Doppler imaging.

*Cardiovascular Magnetic Resonance*

Short-axis cine images extending from the mitral valve to the left ventricular apex were obtained using a balanced steady-state free precession sequence (8-mm parallel slices with 2-mm spacing; temporal resolution ≤45ms). Ventricular volumes, mass and function were quantified using dedicated software (Siemens AG Healthcare Sector, Erlangen, Germany). Papillary muscles and minor trabeculations were included in the volume measurements (and excluded in the left ventricular mass measurements). Left ventricular longitudinal function was determined by measuring the difference in mitral annular displacement between end-systole and end-diastole. The mean value of the lateral and septal insertion sites (4-chamber view) and the anterior and inferior sites (2-chamber view) was used.

For late gadolinium enhancement images both an inversion-recovery fast gradient-echo sequence and a phase-sensitive inversion recovery sequence were performed in two phase-encoding directions to differentiate true late enhancement from artefact. The inversion time was optimized to achieve satisfactory nulling of the myocardium. The distribution of mid-wall fibrosis was described according to the standard 17-segment model recommended by the American College of Cardiology/American Heart Association.

Extracellular expansion of the myocardium was assessed using the Modified Look-Locker Inversion-recovery (flip angle 35°; minimum TI 100 ms; TI increment of 80 ms; time delay of 150 ms; heart beat acquisition scheme of 3-3-5) with built-in motion correction. A gradient echo field map and associated shim were performed to minimize off-frequency artefact. The extracellular volume (ECV) fraction was calculated according to: ECV fraction = partition coefficient x [1-hematocrit], where partition coefficient = [∆R1_myocardium_/∆R1_blood-pool_] and ∆R1 = (1/post-contrast T1-1/native T1). Hematocrit was sampled at the time of cardiovascular magnetic resonance.

*Histological Validation of Myocardial Fibrosis*

The biopsies were fixed in 10% buffered formalin, embedded in paraffin and stained with picrosirius red. An average of eight regions for each sample were analysed using an automated segmentation tool (Image-pro plus 7, Rockville, MD, USA) to quantify myocardial fibrosis (the area stained with picrosirius red as a percentage of the total myocardial area).

*Left Ventricular Hypertrophy*

Using age- and sex-specific thresholds of left ventricular mass index, 89 (54%) patients with aortic stenosis had left ventricular hypertrophy (6 of the 34 with mild aortic stenosis (18%), 24 of the 45 with moderate disease (54%) and 59 of the 87 patients (68%) with severe stenosis). None of the healthy volunteers had left ventricular hypertrophy. Although there was a greater proportion of subjects with hypertension in the aortic stenosis group, the treated blood pressures were similar.

**RESULTS**

**Supplementary Table 1. Aortic stenosis population stratified into tertiles of ECV fraction.**

Although significant differences exist across the tertiles for most measures, ECV fraction was unrelated to aortic stenosis severity.

|  | **Tertile 1**  **(n=54)** | **Tertile 2**  **(n=53)** | **Tertile 3**  **(n=54)** | **P value** |
| --- | --- | --- | --- | --- |
| **Age** | 68 [61, 72] | 71 [65, 75] | 73 [66, 78] | 0.025 |
| **Male gender, n (%)** | 43 (80) | 37 (70) | 32 (60) | 0.07 |
| **Echo** |  |  |  |  |
| **Peak aortic jet velocity, m/s** | 3.74±0.84 | 3.73±0.77 | 3.98±1.1 | 0.30 |
| **Aortic valve area, cm^2^** | 0.96±0.35 | 1.00±0.41 | 0.91±0.31 | 0.58 |
| **Mean AV pressure gradient, mmHg** | 33±15 | 32±14 | 38±25 | 0.29 |
| **- Mild aortic stenosis, n** | 11 | 12 | 11 |  |
| **- Moderate aortic stenosis, n** | 14 | 17 | 14 |  |
| **- Severe aortic stenosis, n** | 29 | 24 | 29 |  |
| **Mean E/e’ ratio** | 12.2±4.7 | 13.7±5.5 | 17.8±10.3 | 0.0003 |
| **CMR** |  |  |  |  |
| **Longitudinal function, mm** | 12.7±2.8 | 12.7±2.8 | 11.4±3.1 | 0.025 |
| **Ejection fraction, %** | 67 [63, 71] | 68 [63, 72] | 67 [63, 71] | 0.87 |
| **Left ventricular mass (indexed), g/m^2^** | 82±14 | 87±20 | 97±26 | 0.002 |
| **Mid wall fibrosis, n (%)** | 1 (2) | 13 (25) | 30 (56) | <0.0001 |
| **Biomarkers** |  |  |  |  |
| **Natural log (hs troponin I)** | 1.6 [1.3, 2.2] | 1.7 [1.3, 2.3] | 2.4 [1.4, 3.4] | 0.002 |
| **Natural log (BNP)** | 2.7 [1.6, 3.7] | 3.3 [2.4, 3.8] | 4.0 [2.9, 4.8] | <0.0001 |
| **Functional Status** |  |  |  |  |
| **Six minute walk test, m** | 420 [360, 455] | 390 [340, 450] | 380 [290, 430] | 0.02 |
| **OUTCOMES** |  |  |  |  |
| **All-cause mortality, n** | 2 | 1 | 11 | - |
| **Mortality rate (per 1000 patient-years)** | 12 | 6 | 78 | 0.0006 |
| **Aortic stenosis-related mortality, n** | 0 | 1 | 9 | - |

AV: Aortic valve, BNP: Brain natriuretic peptide, hs: high sensitivity

**Supplementary Table 2. Aortic stenosis population stratified into tertiles of LV mass index.**

Unlike the tertiles of iECV there was no significant differences in diastolic function, BNP or all-cause mortality across these groups

|  | **Tertile 1**  **(n=55)** | **Tertile 2**  **(n=56)** | **Tertile 3**  **(n=55)** | **P value** |
| --- | --- | --- | --- | --- |
| **Age** | 70 [63, 77] | 70 [65, 74] | 71 [63, 77] | 0.44 |
| **Male gender, n (%)** | 25 (45) | 43 (77) | 47 (85) | <0.0001 |
| **Echo** |  |  |  |  |
| **Peak aortic jet velocity, m/s** | 3.39±0.77 | 3.77±0.74 | 4.35±0.92 | <0.0001 |
| **Aortic valve area, cm^2^** | 1.02±0.37 | 0.95±0.36 | 0.88±0.33 | 0.12 |
| **Mean AV pressure gradient, mmHg** | 26.8 ±13.6 | 32.3±13.0 | 44.8±23.1 | <0.0001 |
| **- Mild aortic stenosis, n** | 23 | 8 | 3 |  |
| **- Moderate aortic stenosis, n** | 14 | 19 | 12 |  |
| **- Severe aortic stenosis, n** | 18 | 29 | 40 |  |
| **Mean E/e’ ratio** | 12.2 [10.1, 15.0] | 12.6 [9.5, 17.0] | 13.6 [10.8, 18.0] | 0.28 |
| **CMR** |  |  |  |  |
| **Longitudinal function, mm** | 13.1±2.9 | 12.1±3.1 | 11.5±2.6 | 0.016 |
| **Ejection fraction, %** | 68 [63, 71] | 66 [63, 70] | 66 [62, 71] | 0.54 |
| **Extracellular volume (ECV) fraction, %** | 27.3±2.0 | 27.1±2.3 | 28.8±3.1 | 0.0008 |
| **Mid wall fibrosis, n (%)** | 4 (7) | 10 (18) | 30 (55) | <0.0001 |
| **Biomarkers** |  |  |  |  |
| **Natural log (hs troponin I)** | 1.3 [0.7, 1.6] | 2.2 [1.5, 2.5] | 2.3 [1.8, 3.1] | <0.0001 |
| **Natural log (BNP)** | 3.1 [2.1, 3.7] | 3.1 [2.5, 4.0] | 3.8 [2.6, 4.5] | 0.060 |
| **Functional Status** |  |  |  |  |
| **Six minute walk test, m** | 400 [340, 440] | 390 [340, 430] | 400 [320, 460] | 0.74 |
| **OUTCOMES** |  |  |  |  |
| **All-cause mortality, n** | 2 | 5 | 7 | - |
| **Mortality rate (per 1000 patient-years)** | 12 | 32 | 47 | 0.23 |
| **Aortic stenosis-related mortality, n** | 0 | 5 | 5 | - |

AV: Aortic valve, BNP: Brain natriuretic peptide, hs: high sensitivity

**Supplementary Table 3. Characteristics of Patients with Moderate and Severe Aortic Stenosis Only (Mild Aortic Stenosis Excluded) Stratified According to Indexed Extracellular Volume Thresholds and Presence of Mid-Wall Late Gadolinium Enhancement**

|  | **Normal myocardium**  **N=47** | **ECV expansion**  **N=24** | **Replacement fibrosis**  **N=35** | **P value** |
| --- | --- | --- | --- | --- |
| **Age** | 71 [66, 75] | 71 [66, 75] | 71 [65, 78] | 0.94 |
| **Male gender, n (%)** | 27 (57) | 19 (79) | 28 (80) | 0.047 |
| **Echo** |  |  |  |  |
| **Peak aortic jet velocity, m/s** | 3.99±0.59 | 4.17±0.80 | 4.31±0.88 | 0.17 |
| **Mean AV pressure gradient, mmHg** | 36.6±11.2 | 41.0±20.0 | 42.4±23.4 | 0.32 |
| **Aortic valve area, cm^2^** | 0.82±0.21 | 0.85±0.21 | 0.83±0.22 | 0.90 |
| - **Moderate aortic stenosis, n** | 18 | 10 | 11 |  |
| - **Severe aortic stenosis, n** | 29 | 14 | 24 |  |
| **Mean E/e’ ratio** | 14.1±9.0 | 14.1±4.6 | 16.3±6.5 | 0.37 |
| **CMR** |  |  |  |  |
| **Left ventricular mass (indexed), g/m^2^** | 76±12 | 97±12 | 108±24 | <0.0001 |
| **Ejection fraction (%)** | 68 [64, 72] | 66 [63, 74] | 67 [64, 72] | 0.49 |
| **Longitudinal systolic function, mm** | 12.9±3.0 | 12.1±2.0 | 11.3±3.1 | 0.042 |
| **Indexed extracellular volume (iECV), mL/m^2^** | 18.7±2.3 | 25.7±3.3 | 30.5±8.3 | <0.0001 |
| **Biomarkers** |  |  |  |  |
| **Natural log (hs troponin I)** | 1.68±1.03 | 2.00±0.81 | 2.58±0.90 | 0.0003 |
| **Natural log (BNP)** | 3.08±1.07 | 3.15±0.84 | 3.42±1.11 | 0.39 |
| **Functional Status** |  |  |  |  |
| **Six minute walk test, m** | 400±77 | 383±104 | 356±142 | 0.23 |
| **NYHA class (%)** |  |  |  |  |
| **1** | 17 (36) | 11 (46) | 15 (43) |  |
| **2** | 21 (45) | 6 (25) | 12 (34) |  |
| **3** | 9 (19) | 7 (29) | 5 (14) |  |
| **4** | 0 (0) | 0 (0) | 3 (9) |  |
| **OUTCOMES** |  |  |  |  |
| **All-cause mortality, n** | 2 | 4 | 8 | - |
| **Mortality rate (per 1000 patient-years)** | 12 | 46 | 76 | 0.047 |
| **Aortic-stenosis related mortality, n** | 0 | 4 | 6 | - |
| **AS-related mortality rate (per 1000 patient-years)** | 0 | 46 | 57 | 0.017 |

AV: Aortic valve, BNP: Brain natriuretic peptide, NYHA: New York Heart Association
